# When is the processing of data from medical implants lawful? The legal grounds for processing health-related personal data from ICT implantable medical devices for treatment purposes under EU data protection law

**DOI:** 10.1093/medlaw/fwac038

**Published:** 2022-10-25

**Authors:** Sarita Lindstad, Kaspar Rosager Ludvigsen

**Affiliations:** Formerly—Law School, University of Strathclyde, Glasgow, UK. Now—Graz, Austria; Department of Computer & Information Sciences, University of Strathclyde, Glasgow, UK

**Keywords:** E-privacy, GDPR, healthcare, ICTIMD, privacy, processing

## Abstract

Medicine is one of the biggest use cases for emerging information technologies. Data processing brings huge advantages but forces lawmakers and practitioners to balance between privacy, autonomy, accessibility, and functionality. ICT-connected Implantable Medical Devices plant themselves firmly between traditional medical equipment and software that processes health-related personal data, and these implants face many data management challenges. It is essential that healthcare providers and others can identify and understand the legal grounds they rely on to process data. The European Union is currently updating its framework, and the special provisions in the GDPR, the current ePrivacy Directive, and the coming ePrivacy Regulation all provide enhanced thresholds for processing data. This article provides an overview and explanation of the applicability of the rules and the legal grounds for processing data. We find that only a cumulative application of the GDPR and the ePrivacy rules ensure adequate protection of this data and present the legal grounds for processing in these cases. We discuss the challenges in obtaining and maintaining valid consent and necessity as a legal ground for processing and offer use case-specific discussions of the role of consent long-term and the lack of an adequate ‘vital interest’ exception in the ePrivacy rules.

## I. INTRODUCTION

Medicine is an emerging field for information communication technologies (ICT). Data processing brings significant advantages, and medical technologies develop at record speeds. ICT-connected Implantable Medical Devices (ICTIMD) plant themselves firmly between traditional medical equipment and software processing health-related personal data. ICTIMD are medical devices^1^ implanted in the human body with software capable of communicating and transferring data to external devices.^2^ They allow healthcare providers to monitor the patient’s condition without being physically present and help medical industries go from reactive to predictive and proactive models of care.^3^

However, the rapid technological development is a two-edged sword, forcing lawmakers and practitioners to balance between privacy, data protection, autonomy, and accessibility. ICTIMD rely on the processing of data on a massive scale, and while they face many of the same data management challenges as other fields, there are some major distinguishing factors.^4^ Health data is one of the most sensitive types of personal data, and the impact of a data breach can have enormous consequences.^5^ ICTIMDs are also, in contrast to most other devices, collecting data automatically and constantly from sensors implanted in human subjects.^6^ The end-user and data subject, the patient, does not have the freedom to leave the device at home. These devices form a particularly sensitive part of the private sphere of the users, demanding high data protection standards.^7^

The European Union (EU) is in the process of updating its privacy and data protection framework. Having replaced the Data Protection Directive (DPD)^8^ with the General Data Protection Regulation (GDPR),^9^ the complimenting ePrivacy directive (PECD)^10^ will eventually be replaced by an ePrivacy Regulation (EPR)^11^ and future additional legislation. These instruments together implement enhanced thresholds for processing health data from terminal equipment. For efficient data protection, it is vital that all the actors in the value chain, the healthcare providers, and the patients can identify and understand the lawful grounds available for processing. Our sections II and III start by clarifying the applicability of the rules and provide an overview of the legal grounds for processing from ICTIMD. Sections IV and V dive deeper into consent and necessity as legal grounds for processing ICTIMD data before section VI discusses the framework’s suitability for ICTIMD processing.

The article will focus on processing enabling medical treatment and exclude processing for research purposes or other public interests. It will be limited to data protection law and will not cover law enforcement access, criminal law issues of illegal access, product liability law, or health law specifically.^12^

## II. APPLYING THE GDPR AND THE EPRIVACY RULES TO ICTIMD ENVIRONMENTS

### A. ICTIMD environments—an introduction

ICTIMD is a diverse group of devices and technologies, but they all share that they are implanted into the human body to support essential functions, through for example monitoring and securing the patient’s heart rate or making sure the insulin levels are stable.^13^All the devices are by themselves small computers, complete with computing power and temporary memory, but due to their environment and size, they typically have little to no cybersecurity enabled. Besides being able to distribute data through short-range technologies to the patient’s phone or the hospital’s computers, the ICTIMD typically also enables remote and continuous monitoring from, eg the patient’s home to the hospital, often through a cloud service as shown in the example below.

We will dive deeper into the relevant parts of the systems in the following discussion of the applicability of the GDPR and the EPR.

### B. The GDPR

The new Medical Device Regulation (MDR) clarifies that the GDPR applies to processing of data generated by medical devices.^14^ According to GDPR Article 1(1), it concerns the protection of natural persons regarding *the processing of personal data.* ‘Personal data’ includes ‘any information relating to an identified or identifiable natural person (“data subject”)’.^15^ The wording ‘any information’ implies a broad scope. The tipping point is whether the person is ‘identifiable’. The GDPR defines an identifiable person as

one who can be identified, *directly or indirectly*, in particular by reference to an identifier such as a name, an identification number, location data, an online identifier or to one or more factors specific to the physical, physiological, genetic, mental, economic, cultural or social identity of that natural person.^16^

Therefore, information about an individual such as their name, age, and medical condition stored on the ICTIMD is personal data.^17^ The GDPR does not apply to anonymous information,^18^ but true anonymisation is hard, especially in the healthcare sector.^19^ Without a direct link between the data subject and the data, the conclusion depends on whether it is ‘reasonably likely to be used’ to identify the person directly or indirectly.^20^ This calls for consideration of all objective factors, ia the costs and the amount of time required for identification.^21^ The data subject may for example be associated with online identifiers provided by the device, applications, protocols, or other identifiers such as Radio Frequency Identification (RFID) tags,^22^ one of the root technologies in ICTIMD.^23^ The implant ID number may also be linked to a back-end database containing information about the individual.^24^ Following this, most ICTIMD-generated data will qualify as ‘personal data’ triggering the application of the GDPR.

Furthermore, ‘data concerning health’ falls within ‘special categories of personal data’, regulated in the GDPR Article 9. According to Article 4(15), ‘Data concerning health’ means ‘personal data related to the physical or mental health of a natural person, including the provision of healthcare services, which reveal information about his or her health status.’ Pursuant to Recital 35, this also includes:

- information collected during the registration,
- a number or other piece of data assigned to a person to identify their health data,
- information from testing or examination, and
- information on a disease, disability, disease risk, medical history, clinical treatment, or the physiological or biomedical state of the subject.

The wording and Recitals entail a wide interpretation of the term, which is consistent with the practice of the European Court of Justice (CJEU) and the purpose of the Regulation.^25^ The definition is independent of the information source and includes originally non-medical data and metadata^26^ when they *in combination* or because of the context say something about the person’s health.^27^ Due to the nature and purpose of ICTIMD, most of the data generated will be ‘data concerning health’. Data about the patient’s lifestyle, environment, and family history are key to modern personalised healthcare.^28^ Together or in a particular context, much of this data may say something about the subject’s health, like buying or using blood glucose metres may reveal information about a diabetes diagnosis.^29^

The GDPR applies to the *processing* of this data. Article 4(2) defines the term ‘processing’ widely, including ‘*any* operation or set of operations performed upon personal data’. The wording covers anything that is done to or with personal data. To minimise the risk of circumvention, the Regulation is technology-neutral.^30^ Simplified, ICTIMD involves the processing of personal data in two ways:

1. The data may be directly stored and communicated through the implant, or
2. by combining information available on the implant, like a unique identifier, with data stored somewhere else, eg in a database.^31^

Complicating the matter, the provisions wording ‘set of operations’ illustrates that *various* operations may constitute *one* processing of personal data.^32^ To identify and fulfil the requirements for lawful processing, it is necessary to distinguish a set of processing activities constituting one processing from others. The GDPR does not solve this question explicitly, but one hint may lie in its system. The Regulation separates the need for new legal bases for processing based on the scope and limits of the *purpose* of the processing.^33^ This means that there is no need to secure a separate legal basis for every processing activity serving the same purpose. Thus, one may argue that it is per the Regulation’s system and efficiency to let the realisation of the purpose of the processing define the activities subject to one processing action.^34^ However, this interpretation is limited to the legal grounds for processing in its narrow sense. In contrast, a single Data Privacy Impact Assessment (DPIA) may address a set of *similar* processing actions resulting in *similar risks* (emphasis added).^35^ Conclusively, the limits of one processing depend on individual assessments in each case. In an ICTIMD context, a specific care pathway, an ambulatory setting, or an examination of a patient may contain a set of operations with both similar risks and purposes, possibly constituting one processing action for a single DPIA and legal ground for processing.^36^

### C. The PECD and EPR

Data processing may fall within the material scope of both the GDPR and the PECD or EPR at the same time.^37^ The PECD was adopted as a complement to the DPD to regulate the electronic communication sector.^38^ According to PECD Article 3(1), it applies to ‘the processing of personal data in connection with the provision of publicly available electronic communications services in public communications networks …, including public communications networks supporting data collection and identification devices.’ As the GDPR has replaced the DPD, the EU is updating the ePrivacy rules accordingly and has provided a proposal for an EPR. Pursuant to the EPR Article 2, it applies to ‘the processing of electronic communications data carried out in connection with the provision and the use of electronic communications services, and to information related to the terminal equipment of end-users’, both with an exception for ‘electronic communications services which are not publicly available’.

The table below provides an overview and comparison of the material scope of the PECD and the EPR. Many of the key entry requirements are similar. In the following, we will therefore assess the two instruments collectively. It is unlikely that the spirit of the PECD and the current EPR will change long term, see Table 1.

**Table 1.** The scope of the PECD and the EPR compared

PECD		EPR	
In general: ‘Personal data’	Article 5(3): ‘information stored in the terminal equipment of a user’	‘Electronic communications data’	‘Information related to the terminal equipment of end-users’
‘Electronic communications services’		‘Electronic communications services’	
‘Electronic communications networks’		‘Communications networks’ are important both as part of the definition of ‘electronic communication service’ and ‘terminal equipment’	
‘Publicly available’		‘Publicly available’	

The PECD is generally only applicable to the processing of ‘personal data.’ Under PECD Article 2 and EPR Article 4(1)(b), unless otherwise provided, the definitions of the GDPR and the European Electronic Communications Code (EECC) apply.^39^ Therefore, for the definitions of ‘personal data’ and ‘processing,’ see the assessments in section II A of this article. The EPR widens the scope and covers ‘electronic communications data’.^40^ This refers to both electronic communications content and metadata.^41^ ‘Content’ includes text, voice, videos, images, and sound, while ‘metadata’ refers to data processed to transmit, distribute, or exchange the content, ia, websites visited as well as geographical location.^42^

Both the processing in the PECD and the EPR must be carried out in connection with the provision and use of a publicly available ‘electronic communications service.’ Neither the PECD nor the EPR defines this. The EECC Article 2(4) reflects the concept of ‘service’ in Articles 56 and 57 TFEU,^43^ and defines ‘electronic communications service’ as:

a service normally provided for *remuneration* via electronic communications networks, which encompasses, …, the following types of services: (a) ‘internet access service’ … (b) interpersonal communications service; and (c) services consisting wholly or mainly in the conveyance of signals such as transmission services used for the provision of machine-to-machine services.

‘Remuneration’ is interpreted widely by the CJEU. The essential characteristic is that it constitutes *consideration* for the service in question,^44^ but it does not have to be profitable.^45^ The remuneration does not have to originate from the person benefiting from the service.^46^ In an ICTIMD environment, several applications enable data transmission to the patient’s doctor. This function is typically provided for remuneration, and despite the patient not always covering the costs themselves, it usually constitutes an electronic communication service.

Furthermore, the EPR applies to ‘information related to the terminal equipment of end-users,’ and the PECD Article 5(3) sets forth special conditions for storing and accessing such information. The EPR Article 8 aligns the terminology with the GDPR and regulates the use of *processing* and storage capabilities and collection of information from terminal equipment. As opposed to the GDPR and the PECD in general, PECD Article 5(3) and the EPR do not only apply to personal information but *any kind of information* stored on the terminal equipment of the end-user.^47^ The EPR and the EECC both refer to the definition of ‘terminal equipment’ in Article 1(1) of Directive 2008/63/EC.^48^ According to this, ‘terminal equipment’ means ‘equipment directly or indirectly connected to the interface of a public telecommunications network to send, process or receive information ….’ This definition is broadly formulated and includes not only personal computers or other typical user terminals such as mobile phones, but equally applies to RFID, chip cards, and intelligent implants.^49^

The terminal equipment or communications service must be connected to a publicly available electronic communications network. Under the EECC Article 2(1), ‘electronic communications network’ means transmission systems permitting the conveyance of signals, irrespective of the type of information conveyed. As recognised in the EPR Recital 12, connected devices increasingly communicate through electronic communications networks. By including the formulation ‘communications networks supporting data collection and identification devices’ in the PECD Article 3, the Commission wished to clarify that the PECD should apply to RFID devices connected to public communications networks.^50^ There is a broad range of wireless technologies and protocols in use for medical applications, of which RFID and machine-to-machine technologies are fundamental.^51^

The EECC Article 2(8) defines a ‘publicly available’ electronic communications network as a network wholly or mainly used to provide publicly available electronic communications services.^52^ The EPR Recital 13 as amended in January 2021,^53^ prescribes that to the extent that the networks are provided to an *undefined group* of end-users, regardless of whether these networks are secured with passwords or not, the confidentiality of the communications should be protected. Closed groups of end-users such as home or corporate networks or networks where access is limited to a *pre-defined group* of end-users are not covered.^54^

Letting the distinction depend on the group of end-users with access to the network or service is in line with the definition used by technical sciences, defining a ‘public network’ as a communications network that anyone can use.^55^ The distinction is challenging to use in practice as services are increasingly becoming a mixture of private and public elements.^56^ This is especially true when monitoring the patient remotely. Insofar the processing is happening through a treatment facility’s closed network or the patient’s home network, the communication will fall outside the definition of ‘public network’. However, once the data leaves this sphere, it is usually communicated further through a cloud service on a public network where the care unit can access it through an extranet connection.^57^ The implant will also have to follow the patient outside the range of their private home networks, connecting via publicly available cellular communication networks.

There are remedies complicating this picture, such as overlay networks,^58^ encryption, and fog computing^59^ at gateways.^60^ In most cases, ICTIMD devices will to some extent communicate through publicly available networks. The EPR Recital 13 clarifies that the provisions regarding the protection of terminal equipment information also apply in the case of equipment connected to a closed group network which *in turn* is connected to a public electronic communications network. The European Data Protection Supervisor (EDPS) highlights that the coming EPR should provide the same level of protection for communications stored on other equipment than user terminals, for example, in mailboxes operated by a service provider or cloud storage used as part of the communications service.^61^

Therefore, most ICTIMD cases will be covered by the PECD and the EPR and qualify as the ‘terminal equipment of a user’.

### D. The subjects of the obligations

Under the GDPR, the duty to demonstrate a legal ground for processing lies on the data ‘controllers.’ Pursuant to GDPR Article 4(7), ‘controller’ means a natural or legal person ‘which, alone or jointly with others, *determines* the purposes and means of the processing of personal data’. In an ICTIMD context, the care providers (hospitals and clinics) determine the means and purpose of the processing, for example, to monitor the patient’s heart rate for diagnosis. They share the responsibility depending on their role in the decision-making.^62^ The manufacturers of medical devices also often qualify as controllers as they may have modified the operating system or installed software determining its functionality.^63^

**Figure 1.**
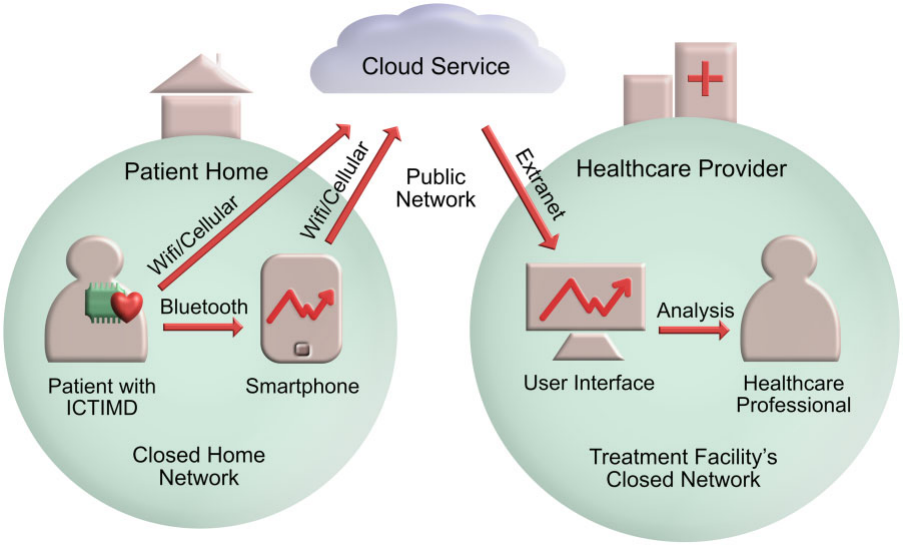
A typical remote patient monitoring system. Figure inspiration from Oskari Koskimies, ‘The Future of Remote Patient Monitoring Is in Artificial Intelligence’ (MEDDEVOPS, 2019) <https://meddevops.blog/2019/10/09/the-future-of-remote-patient-monitoring-is-in-artificial-intelligence/> accessed 26 July 2022.

Others may also process ICTIMD data. According to the GDPR Article 29, a ‘*processor* and any person acting under the *authority* of the controller or of the processor’ may process the data when instructed by the controller or required by law. A ‘processor’ is defined as a separate person or entity processing personal data ‘on behalf of the controller’,^64^ for example, the cloud service provider in Figure 1.^65^ A person acting under the ‘authority of the controller or processor’ will typically encompass employees *identifiable* with the controller or processor entity,^66^ like an assistant at the hospital. Moreover, the stakeholders of an IoT ecosystem include different suppliers and integrators.^67^ As far as they are not legally identifiable with the controller or processor, they constitute third parties to be regulated by contract. The controller does, however, retain its role in determining the purpose and means of processing.^68^

While the primary duties in the GDPR are on the controllers, the requirements in the PECD Article 5(3) concern *all stakeholders* who want to store or gain access to the raw data in the terminal equipment.^69^ It applies without regard to the nature of the entity, whether public or private, a single individual or a major corporation, or whether it is a data controller, data processor, or a third party.^70^ Following this, the responsibility for obtaining consent in the EPR should, according to the January amendments Recital 20aaa, apply to the entity that uses the processing and storage capabilities or collects information from the terminal equipment. This includes information society service or network providers.^71^ Such entities may ask another party to obtain consent on their behalf.^72^

## III. MAPPING THE LEGAL GROUNDS FOR PROCESSING ICTIMD DATA

### A. The legal grounds for processing according to the GDPR

For data to be processed lawfully, the processing must comply with one of the legal grounds for processing listed in GDPR Article 6(1).^73^ However, data generated by ICTIMD qualifies as ‘special categories of personal data’ covered by GDPR Article 9. It provides a general prohibition for processing these kinds of data together with a list of exceptions.

The relationship between GDPR Articles 6 and 9 has been a topic of much discussion.^74^ Whether the exceptions in Article 9 are to be understood as its own list of legal grounds for processing, or if the two articles are meant to be applied cumulatively is not explicitly solved in the GDPR.^75^ But a cumulative application has become mainstream legal doctrine.^76^ In addition to the specifics of Article 9, the general principles and other rules of the GDPR, including Article 6(1) apply.^77^ Such an interpretation is in line with the purpose and efficiency of the Regulation. If Article 9 was to provide a sufficient legal basis alone, it could in some situations lead to a lower level of protection for special category data than for others.^78^

Under GDPR Article 9(1), processing data concerning health is generally prohibited and only allowed in exceptional cases listed in Article 9(2). The most relevant exceptions for processing in the field of ICTIMD for treatment purposes are when:

- ‘the data subject has given explicit consent’ (a),
- ‘processing is necessary to protect the vital interests of the data subject where the data subject is physically or legally incapable of giving consent’ (c), and
- ‘processing is necessary for the purposes of preventive or occupational medicine’, medical diagnosis, the provision of health or social care or treatment based on ‘Union or Member State law or pursuant to contract with a health professional’ (h).

According to the GDPR Article 5(1)(b), data cannot be further processed in a manner that is incompatible with the given purposes. Pursuant to Article 6(4), this depends on a case-by-case assessment of the connection to the primary purpose, the context, the nature of the personal data, and the possible consequences for the data subject. Due to the sensitivity of the data and situation, this threshold will generally be high in ICTIMD contexts.

### B. The legal grounds for processing according to the ePrivacy provisions

The PECD and the EPR both operate with different categories of data entitled to different levels of confidentiality.^79^ While the data related to ICTIMD may include both traffic- and location data according to PECD Articles 6 and 9,^80^ and electronic communications content and metadata regulated in EPR Article 6, the discussions above have shown that most of these devices qualify as terminal equipment regulated in PECD Article 5(3) and EPR Article 8. These Articles concern all stakeholders and are meant to limit the lawfulness of the processing when it includes terminal equipment, regardless of the nature of the information.^81^

Like the structure of the GDPR Article 9, the PECD Article 5(3) and the EPR Article 8 generally prohibit the storage of and access to information on the terminal equipment before providing some specific exceptions. The exceptions of particular interest for enabling medical treatment in the field of ICTIMD are when:

- the end-user has given his or her consent, or
- it is strictly necessary for providing an information society service requested by the end-user.

The 2021 amendment to the EPR Article 8(1)(f) also suggests adding new legal grounds of interest in ICTIMD contexts, hereunder when it is necessary to locate terminal equipment because an end-user emergency communication.

If the access concerns data already imported from the device and stored on the server of, eg a device manufacturer, it is no longer subject to the PECD or EPR but to the provisions of the GDPR on the legitimacy of further processing.^82^ The January amendments of the EPR Article 8(1)(g1) suggest extending the further processing protection to *anonymised* data not covered by the GDPR.

### C. The relationship between the GDPR and the ePrivacy instruments

The first data processing from the ICTIMD might be regulated by both the GDPR Article 9 and the PECD Article 5(3) or EPR Article 8. According to the PECD Article 1(2) and the EPR Article 1(3), the instruments aim to ‘particularise and complement’ the GDPR regarding the processing of personal data in the electronic communication sector. Pursuant to the principle of lex specialis,^83^ in situations where the PECD/EPR ‘particularises’ the rules of the GDPR, the specific provisions of the PECD/EPR shall take precedence over the general provisions of the GDPR.^84^ On the other hand, any processing of personal data not specifically governed by the PECD/EPR remains subject to the provisions of the GDPR.^85^ To define how far the derogations go, careful analysis of the facts in each case is necessary, particularly where there are several types of processing.^86^

To the extent that the information stored in the end-user’s device constitutes personal data, the PECD Article 5(3) and EPR Article 8 shall restrict the GDPR catalogue over legal grounds for processing.^87^ The sector-specific rules should not leave the data subject less protected than under the GDPR.^88^ While the PECD or the EPR limits the list of available legal grounds processing, the processing of personal data must still have a legal basis under the GDPR to be lawful.^89^ Thus, neither isolated compliance with the GDPR nor with the PECD Article 5(3) or the EPR Article 8 is sufficient for legitimate processing from ICTIMD.^90^ Similar to the mentioned relationship between GDPR Articles 6 and 9, the ePrivacy rules must also be cumulatively applied to ensure the full efficiency of the system.^91^

Together, GDPR Article 9 and PECD Article 5(3) or EPR Article 8 drastically restrict the list of relevant legal grounds for processing from ICTIMD. Table 2 gives an overview of the remaining grounds.

**Table 2.** Overview of the legal grounds for processing data from ICTIMD

	PECD Article 5(3), EPR Article 8	GDPR Article 9
Consent	Informed consent in acc. with GDPR	Informed, explicit consent
Necessity	Processing is strictly necessary to provide an information society service explicitly requested by the user.	Processing is necessary to protect the data subject’s vital interests where the data subject is physically or legally incapable of giving consent.
		Processing is necessary for the purposes of preventive or occupational medicine, medical diagnosis, the provision of health or social care or treatment based on Union or MS law or pursuant to contract with a health professional.

There are two types of closely related, practical grounds for processing that are interesting to discuss for both frameworks: *consent* and *necessity*. We will elaborate in the following two sections.

## IV. CONSENT AS LEGAL GROUND FOR PROCESSING ICTIMD DATA

### A. The conditions for valid consent

Consent is rooted in self-determination, integrity, and designed to foster choice and formalisation of agreement.^92^ The standard of consent in the PECD and EPR is the same as in the GDPR.^93^ The EPR Article 9 transfers the definition of consent in Articles 4(11) and 7 of the GDPR to the EPR. Following the cumulative relationship between the GDPR provisions, consent for processing health-related data must comply with all conditions found in GDPR Article 4(11), 6(1)(a), 7, and 9(2)(a). Article 4(11) defines consent as

any *freely given*, *specific*, *informed* and *unambiguous indication* of the data subject’s wishes by which he or she, by a *statement or by a clear affirmative action*, signifies agreement to the processing of personal data.

For the special categories of data under Article 9(2)(a), it must be ‘*explicit*’ for ‘one or more specified purposes’. The following chapters will dive deeper into these requirements and discuss issues in ICTIMD environments.

### B. Freely given

Consent must be ‘freely given’, which means that the data subject has a genuine choice and can refuse or withdraw consent without detriment.^94^ This calls for a comprehensive assessment of each situation. Where the data subject is in a particularly vulnerable position, or there is a clear imbalance between the data subject and the controller, valid consent may be unlikely.^95^ In ICTIMD contexts, the possibility to renounce services or features is often more a theoretical alternative. Data processing becomes a necessary ‘by-product’ of receiving healthcare, a fundamental human right.^96^ According to Article 29 Data Protection Working Party (WP29), consent is not freely given if it is given under the threat of non-treatment or lower quality medical treatment.^97^ If a health professional has to process personal data as an unavoidable consequence of the medical situation, it is misleading if he seeks to legitimise this processing through consent.^98^

The vulnerable situation of the patient and their position in relation to a manufacturer or healthcare professional might create an imbalance between the data subject and the controller. Imbalance might arise when the patient is not in good health or by situations of institutional or hierarchical dependencies.^99^ The inequality in knowledge makes the patients dependent on the doctor to understand their situation. Transparency and information can compensate for an imbalance due to lack of knowledge, but this alone is not enough to legitimise the processing.^100^

### C. Informed

Information is an inevitable part of genuine consent.^101^ Unless the patient’s decision builds on sufficient information about all alternatives, the consent given is reduced to a mere formality.^102^ Under Article 7(2), the information should be presented to users in an ‘intelligible and easily accessible form, using clear and plain language’. This means that a user can *easily* determine the consequences of the consent and that the information is ‘clearly comprehensible and sufficiently detailed’.^103^

According to the GDPR Articles 13 and 14 and Recital 39, the data subject should be aware of, ia, the origin of the data, the identity of the controller, the categories of recipients, the purpose and logic of the processing operations as well as the timeframe, risks, rules, safeguards, and rights concerning the processing. Moreover, the controller should provide any further information necessary to ensure fair and transparent processing considering the specific circumstances and context.^104^ Of particular interest for ICTIMD is the duty to inform about the existence of automated decision-making, including profiling in Article 22(1) and (4). In those cases, ‘meaningful information’ should be provided about ‘the logic involved, as well as the significance and the envisaged consequences of such processing for the data subject.’^105^ What constitutes ‘meaningful information’ will depend on the situation. Per GDPR Articles 5, 7(2), 13, and 14, the information should include the reasons and the basis for the decision in a way that the data subject can understand.^106^

Conclusively, valid consent requires a thorough understanding of the data journey. This is often hard to obtain in complex ICTIMD systems.^107^ The OECD and WP29 have noted that the uses of personal data are becoming increasingly complex and non-transparent to individuals.^108^ The processing of ICTIMD data usually relies on the coordinated intervention of several stakeholders involved with various purposes and degrees of control. There may be multiple care providers working on the same patient, and a variety of stakeholders are included to provide functionality or easy-to-use control interfaces.^109^ The number of actors leads to lengthy and complex consent forms, making them harder for patients to understand. To illustrate this, The Norwegian Consumer Council found that the average word count of the terms of use of blood glucose metres and blood pressure apps stands at 6.653.^110^

To this, WP29’s position states that controllers ‘should separately spell out in unambiguous language what the most important consequences of the processing will be’.^111^ For automated decision-making processes, some argue that the quality of explanations might not be an adequate safeguard alone.^112^ They suggest implementing additional tools like algorithmic DPIA based on Artificial Intelligence (AI) to complement explanations.^113^ Increased use of AI for DPIA is likely to warrant enhanced transparency and legitimate attempts to provide ‘meaningful information.’^114^ The General Secretariat of the Council suggests adding DPIA as a requirement in addition to consent for providers of electronic communications networks and services.^115^

### D. Specified purposes

Pursuant to the GDPR Article 9(2)(a) together with 5(1)(b), the data subject must consent explicitly for one or more specific, explicit, and legitimate purposes before processing data. The purpose limitation combined with explicit consent serves as safeguards against widening or blurring of the purposes for data processing.^116^ The consent should cover all processing activities carried out for the same purpose, and when the processing has several purposes, consent should be secured for all of them.^117^ A vague or general purpose, such as ‘improving user experience’ or ‘IT-security purposes’, will not be specific enough.^118^ A general agreement on collection and use of medical data in electronic health records for any future use would also not meet the threshold.^119^

Most ICTIMD have a specific purpose in mind. A pacemaker, for example, focusses on heart rhythm. However, when the data is exported, this might add some purposes to the list, eg remote patient monitoring or device maintenance, necessary to specify in the consent form. As big data now allows a so-called discovery-driven approach,^120^ there is a tendency towards broader rather than specific purposes.^121^ Given the open-ended character of these technologies, data controllers find it harder to specify why they are processing personal data.^122^ To secure specific and informed consent, some suggest ‘dynamic consent’, where the data subject initially gives a broad consent, and then later specify their preferences.^123^

### E. Explicit

The consent must furthermore be ‘explicit’ for the given purposes. The GDPR does not define the term ‘explicit’, but the wording entails that implied consent with opt-out solutions is unacceptable. ‘Explicit’ must be more than ‘unambiguous’ consent in Article 6. Several formulations in the preparatory work to the GDPR and the Regulations system show that the distinction between ‘explicit consent’ and ‘consent’ is intentional.^124^ While giving the burden to prove sufficient consent to the controller,^125^ the GDPR does not specify a method or form of demonstration. According to the WP29, ‘where appropriate, the controller could make sure the written statement is signed by the data subject, in order to remove all possible doubt and potential lack of evidence in the future’.^126^ Therefore, while oral statements may express valid consent, the sensitive nature of ICTIMD data calls for written consent.^127^

In a digital context, the data subject may consent by filling in an electronic form, sending an email, or using an electronic signature.^128^ Under the EPR Article 9(2), where technically possible and feasible, the data subject may consent using a software application’s technical settings. The EPR Article 10(2) prescribes that upon installation, the software shall inform the end-user about the privacy settings options and require the end-user to consent to a setting to continue with the installation. Such a system will provide the security of a documented choice and ensure its availability to everyone involved in the care process. A point of possible concern in the ICTIMD context is that the January 2021 amendment’s Article 4a(2a) suggests adding that if the provider cannot identify the data subject, ‘the technical protocol showing that consent was given from the terminal equipment shall be sufficient to demonstrate the consent of the end-user.’ If this is implemented into the final version, the risk of circumvention is increased either through wilful ignorance or deliberately. To protect the end-user’s self-determination in these cases, the General Secretariat of the Council suggests that consent directly expressed by an end-user shall prevail over software settings.^129^

### F. Timing

The GDPR does not explicitly state at what time in the process the data subject must give consent. To foster autonomy, consent must be obtained before the data processing.^130^ This interpretation might seem out of line with the formulation of the information duty in Article 13(1). It demands that the controller ‘at the time when personal data are obtained’ must provide the data subject with all necessary information. A central part of the principle of transparency is that ‘the data subject should be able to determine *in advance* what the scope and consequences of the processing entails.’^131^ In an ICTIMD context, the nature of the situation, the data collected, and the complexity calls for early information and opportunities for questions.

The patient’s situation might require data processing continuously and unnoticed over long periods. The obligation to demonstrate consent exists during the data processing.^132^ The consent is likely to degrade over time as it builds on strict information requirements and concrete circumstances. The GDPR does not specify how long a consent is valid, but the WP29 state that it ‘should be refreshed at *appropriate intervals*’.^133^ It depends on ‘the context, the scope of the original consent and the expectations of the data subject’.^134^ In the EPR, the timeframe has changed several times. Article 9(3) proposes a duty to remind the subjects of data processed according to Article 6(2) and (3)(a) and (b) at intervals of 6 months. The January 2021 draft Article 4a(3) sets this interval to 12 months before the General Secretariat of the Council suggests applying the 12-month interval to all data processing under the Regulation.

For ICTIMD, due to its duration, invasiveness, and sensitivity, there should be ways to ensure that patients remain aware of the transmission of their health data when the treatment via electronic means becomes routine.^135^ Taking the suggested minimum requirements of at least every sixth and twelfth month in the EPR drafts as a reference, the appropriate minimum interval for such sensitive data might be around 6 months. Updating the consent and reminding the patient of the possibility of withdrawing it at periodic intervals is easier through well-developed software-based solutions. For the consent to stay informed throughout the device’s lifetime, any changes to the terms of use should also be announced in advance, giving the patients sufficient opportunity to exercise their rights.^136^

## V. NECESSITY AS LEGAL GROUND FOR PROCESSING ICTIMD DATA

### A. The different necessity grounds in an ICTIMD context

The GDPR and the PECD or EPR also allow processing based on necessity, but none of the frameworks clearly define it. As pointed out by the EDPB, the

concept of necessity has an independent meaning in European Union law, which must reflect the objectives of data protection law. Therefore, it also involves consideration of the fundamental right to privacy and protection of personal data.^137^

Assessing what is ‘necessary’ implies a combined, fact-based assessment of the processing in relation to the purpose.^138^ The GDPR Article 9(2) and PECD Article 5(3) or EPR Article 8 set forth some slightly differing purposes, of which the most relevant in our context is:

- Provision of a service requested by the user
- Medical purposes based on law or contract with a healthcare professional
- Protecting the vital interests of the data subject

Moreover, the GDPR Article 5(1) contains the data minimisation principle. The processing is not ‘necessary’ if there are realistic, less intrusive alternatives.^139^

### B. The provision of a service requested by the user

The GDPR Article 6(1)(b) allows for processing ‘necessary for the performance of a contract to which the data subject is party’. For the electronic communication sector, the PECD Article 5(3) limits this to storage or access when it is strictly necessary to provide an information society service explicitly requested by the subscriber or user to provide the service. The word ‘strictly’ has, without explicit reason, been removed from Article 8 in the EPR. It is still highlighted in its Recital 21 and was reinstated in the equivalent Article 8(1)(c) in the January 2021 amendment.

‘Strictly necessary’ implies a high threshold,^140^ and it is not enough that the storage or access is important. It must be *essential* to provide the service requested by the user from the user’s point of view.^141^ Thus, it does not cover what might be essential for any other uses the controller might wish to make of that data. It does allow what is required to comply with any other legislation that applies to the controller, ia, security requirements.^142^ Moreover, the extent of this exception depends on the scope of the relevant service.

From a user perspective, the central features of an ICTIMD will be its ability to detect, collect and communicate information about a medical issue and treatment. This covers services providing the relevant storage and access to sensor-registered health-related data. However, to enable these features and maintain the security requirements of medical devices, it is also necessary to perform software updates and gather information about the device’s performance. As far as this is strictly necessary and proportionate to provide the ICTIMD service, this should be covered under the PECD Article 5(3) or the EPR Article (8)(1). Recognising this, the January 2021 amendment Article 8(1)(e) adds a separate legal ground for processing to make software updates for security reasons.

### C. Medical purposes based on law or contract with a healthcare professional

For personal, health-related data, GDPR Article 9(2)(h) restricts the above interpretation of the PECD and EPR. It permits processing necessary for medical diagnosis and provision of healthcare, limited to the ‘true needs and the medical relevance’.^143^ The processing must be based on union or Member State (MS) law or a contract with a healthcare professional ‘and subject to the conditions and safeguards referred to in paragraph 3’. The definition of health professionals is left to the MS. According to Article 9(3), the data may only be processed by or under the responsibility of a professional or others subject to the obligation of secrecy under Union or MS law or rules established by competent national bodies.

The primary purpose of most ICTIMD is precisely to provide healthcare, and to establish a connection between the data and a medical need should not be problematic. In everyday healthcare, medical professionals often use Article 9(2)(h) to process data without consent for each operation in a ‘treatment relationship’.^144^ According to WP29 a ‘treatment relationship’ may include the doctor treating the patient and other professionals at the same institution.^145^

Whether any other data controller can process data under this provision, depends on several factors. They will normally not be subject to a national obligation of secrecy themselves and processing must in case happen ‘under the responsibility’ of the physician. The GDPR does not elaborate what is meant by ‘under responsibility’ of the physician. According to Kuner and Georgieva, it includes processing carried out using medical devices or apps, if they are used under the responsibility of such a professional.^146^ Hence, other processing done by an external commercial actor like a manufacturer, is typically not taking place ‘under the responsibility’ of the physician.^147^ The extent of the responsibility of the health professional depends on MS law.

### D. Protecting the vital interests of the data subject

Pursuant to GDPR Articles 6(1)(d) and 9(2)(c), ‘where the data subject is physically or legally incapable of giving consent’, processing can take place as far as necessary to protect the ‘vital interests’ of the data subject. A ‘vital interest’ of a person is an interest that is essential for the data subject’s life.^148^ The wording implies a high threshold, with life and death situations at its core. The PECD does not contain an equivalent vital interest exception, and the EPR only introduces it explicitly for metadata processed within Article 6b(1)(d). The January 2021 version of the proposal adds an explicit opportunity to use the device’s processing and storage capabilities *to locate* terminal equipment when an end-user makes an emergency communication.^149^

From a fundamental human rights perspective, they should allow processing of *content* data from terminal equipment when necessary to save the data subject’s life.^150^ The ePrivacy rules allow what is required to comply with other legislation that applies to the controller, ia, the security requirements.^151^ This interpretation is necessary to keep the EU framework consistent and efficient.^152^ The GDPR Article 32(1)(c) demands that the controller and the processor ensure a level of security appropriate to the risk, including ‘the ability to restore the availability and access to personal data in a *timely manner* in the event of a physical or technical incident’.

ICTIMD could contain health data likely to save a patient’s life in emergencies. Being able to access the information in these situations, hospitals would immediately know how to treat an incoming patient. In such a situation, the personnel will not be able to follow advanced authorisation procedures to obtain control over the device.^153^ Therefore, a vital interest exception is highly practical for the processing of ICTIMD data. Both the WP29 and the EDPS have highlighted that adding such an exception in the ePrivacy instruments is necessary.^154^

## VI. THE SUITABILITY OF THE FRAMEWORK

### A. The fine balances

The legal grounds for processing data are indispensable gatekeepers designed to secure both the data subjects’ data protection rights and leave room for the use of new technologies. The development of the new EPR spurs life into questions about the content of the rules and the necessity of separate legal grounds for processing data in the telecommunications sector. This section assesses the framework’s suitability for ICTIMD processing based on two of its primary goals, balancing fundamental rights and providing predictability.

### B. Human rights perspectives

As an ICTIMD becomes an integrated part of the patient's private sphere, access, and data processing brings several fundamental rights into play. Health data is particularly sensitive, and respect for its confidential nature ‘constitutes one of the fundamental rights protected by the legal order of the European Union’,^155^ in particular through the Charter of Fundamental Rights of the European Union (Charter).^156^ However, the rights are not absolute and may be limited as far as the limitations respect the essence of the rights, are necessary, and meet objectives of general interest.^157^ The rights must be considered in relation to their function in society and balanced against other fundamental rights under the principle of proportionality.^158^

Pursuant to the Charter’s Article 7, the right to privacy provides that ‘everyone has the right to respect for his or her private and family life, home and communications.’ One of its most important objectives is to prevent improper use of personal information.^159^ This has fostered the development of the right to protection of personal data in Article 8, recognising that decision on the publication, sharing, and storage of personal data is part of the individual’s ‘informational self-determination’.^160^ Finally, the right to protection of personal integrity demands respect for ‘the free and informed consent of the person concerned’ in the field of medicine.^161^

The GDPR and PECD or EPR aim at giving integrity, autonomy, and self-determination a central space. However, section IV has shown that ‘freely given’ and sufficiently ‘informed’ consent may be problematic in ICTIMD contexts. EU law operates with an understanding of ‘freely given’ that does not leave much room for using consent as a legal ground for processing data from ICTIMD. While consent has been the main rule in the PECD, the GDPR and the EPR Article 8 have not given it priority over the other legal grounds. According to the EPR Explanatory Memorandum, the implementation of the PECD has not been effective to empower end-users, as they face requests without understanding their meaning.^162^

Many do, however, consider informed consent an essential element of informational self-determination, especially for medical data.^163^ They stress that while the intention of weakening consent as a legal ground for processing may be to shift the burden of privacy protection away from individuals and towards data controllers. The effect will be to weaken fundamental privacy rights of individuals and strengthen the power of data controllers to decide what, how, and when to collect and process data.^164^ They hold that individuals will lose the opportunity to ia make consent conditional, revoke or deny consent for new purposes, be informed of the existence of record-keeping systems, access data, verify the accuracy of one’s data, obtain explanations of the use, and challenge the compliance of data controllers.^165^

Many of these concerns are met in the EU system by demanding transparency in relation to the data subject, and a duty to take ‘every reasonable step’ to keep the data accurate, no matter what legal ground is used.^166^

To fully limit the grounds for data processing in ICTIMD environments to cases of informed consent may conflict with the right to life or the right to health.^167^ The right to life is not only implying a duty not to take away anyone’s life, but also a positive duty to take reasonable steps to protect it. The right to health prescribes that ‘Everyone has the right of access to preventive healthcare and the right to benefit from medical treatment under the conditions established by national laws and practices.’ ICTIMD could contain health data that are likely to save a patient’s life and future health in emergency situations. Furthermore, continuous, discovery-based processing is an indispensable part of everyday healthcare for many patients that might leave consent insufficient and impractical. Thus, there is an inherent need for a well-balanced compromise between data protection and privacy measures on one hand and functionality on the other.

The GDPR provides legal grounds for processing that are useful both in everyday healthcare as well as emergencies while requiring a proportionate level of protection in each case. The PECD is on the other hand lacking a clear vital interest exception. The exceptions for meta and location data in the EPR are likely to come short in case of a medical emergency, when access to the communications *content* is vital for complying with fundamental human rights. Therefore, in our opinion, a vital interest exception also for information related to terminal equipment is necessary for the upcoming EPR.

### C. Predictability

For efficient data protection, it is essential that healthcare providers and other actors in the value chain can easily identify and understand the lawful grounds they may rely on to process data from ICTIMD.^168^ Clear and easily accessible rules lead to high predictability and in turn more robust access to justice. This also makes it key for patients’ trust in ICTIMD systems, and in turn the quality of their medical treatment.

As the GDPR is a non-sector-specific regulation, making clear-cut standards that at the same time give room for the diversity and development of all fields is a challenge. Despite being a regulation binding in its entirety,^169^ the rules must be flexible and leave room for individual application in each case. Therefore, the GDPR’s approach allows controllers room to justify their data processing in situations were relying on the user's consent is not possible. The PECD and EPR, on the other hand, regulate the telecommunications sector specifically. As this is a quite diverse sector in rapid development, the need for flexibility is however still present. To meet real-world requirements, both lobbyists and the industry have recommended aligning the ePrivacy rules and the GDPR by making storage or access lawful if it meets the criteria of the GDPR.^170^ Some go even further and question the need for an EPR in addition to the GDPR at all.^171^ One of the main arguments is that sector-specific data protection legislation may lead to legal uncertainty due to conflicting or unclear provisions.^172^ Also in academic circles, it is known that sector-based approaches frequently suffer from poor calibration and artificial splits of the ‘sectors’.^173^

Others argue that the protection of terminal equipment has characteristics that are not clearly addressed by the GDPR, which is not specifically covering the confidentiality of information on an individual’s device and are in favour of keeping two sets of rules.^174^ As opposed to the GDPR, the PECD Article 5(3) and the EPR Article 8 also cover *any* type of information and directly concern all stakeholders who want to process data from ICTIMD. One may argue that this complementary set of rules ensures a layer of precision necessary to provide predictable and adequate protection.^175^ Also, a study done by the Directorate General for Internal Policies shows that separate ePrivacy rules can improve legal clarity.^176^ They hold that the GDPR contains many general provisions with open norms that are too vague for the situations regulated in the ePrivacy rules.

The GDPR does, however, operate with specific standards for health-related data in Article 9, which are not considered in the PECD and EPR. These are, as shown, necessary to adequately protect health-related data and the patient, especially in crisis situations. The need for such specific rules is also highlighted by the EUs current work on the Regulation of the European Health Data Space (EDHS).^177^ The EDHS builds on the possibilities in the GDPR for a specific EU legislation on the use of personal electronic health data and recognises that uneven implementation of the GDPR by MS creates considerable legal uncertainties in this domain.^178^ Besides establishing specified criteria for processing, the current draft requires the MS to establish digital health authorities to ensure the implementation of the rights and obligations as well as health data access bodies responsible for granting certain accesses.

Conclusively, the ePrivacy rules and the GDPR may be described as specific in two different fields or sectors relevant for processing ICTIMD data. However, interpreting them together may be a complex exercise and the differences between them may lead to insecurities, also in time-sensitive situations. In our opinion, eliminating the illustrated discrepancies between the two sets of rules is of particular importance for predictability in ICTIMD contexts.

## VII. CONCLUDING REMARKS

**Figure 2.**
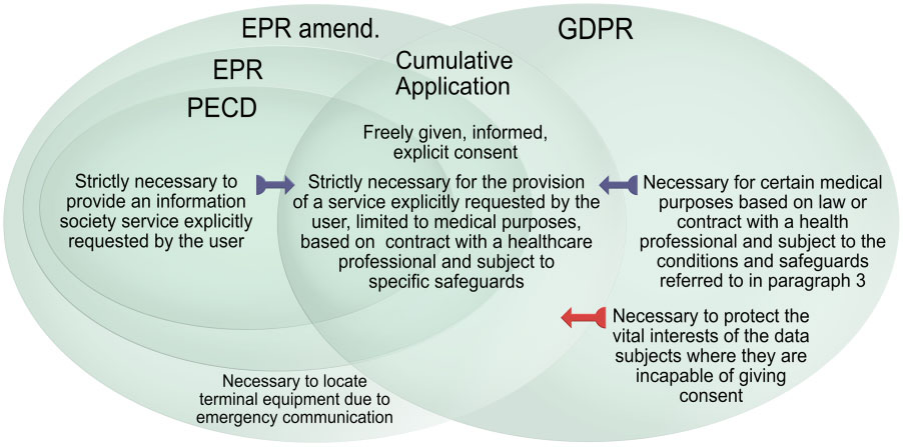
Overview of the cumulative application of the ePrivacy rules and the GDPR.

The legal grounds for processing data in ICTIMD contexts are regulated in the GDPR Articles 6 and 9, as well as the PECD Article 5(3) and the new EPR Article 8. GDPR Article 6 provides the general legal grounds for processing personal data, while Article 9 regulates certain special categories of data, including health-related data. The PECD Article 5(3) and the new EPR Article 8, applies to information related to the terminal equipment of end-users, encompassing most ICTIMDs. In the context of ICTIMD, the GDPR and the ePrivacy rules must be applied cumulatively to ensure efficient protection. As illustrated in Figure 2, GDPR Article 9 and PECD Article 5(3) or EPR Article 8 mutually restrict the list of available legal grounds for processing. To process data lawfully, controllers must first establish that the prohibition on processing in PECD Article 5(3) or EPR Article 8 and GDPR Article 9(1) can be overcome by identifying applicable exceptions. Then they must ensure that one of the lawful grounds for processing in GDPR Article 6 applies and comply with the general principles in Article 5.

To the extent that the patient does not face the threat of non-treatment or lower quality treatment, data may be processed based on freely given, specific, informed, and explicit consent. In practice, sufficiently informed consent is a tricky and high-maintenance legal basis for processing in ICTIMD contexts. One may question whether the EU’s understanding of ‘freely given’ is slightly too restrictive in practice in ICTIMD cases, as external ‘pressure’, strong or weak, will exist in all these situations. The EU has decided that the pressure of one’s health is too big for consent to be valid. While it might be justified to protect the patient extra in these situations, it is, in our opinion, important to recognise the limitations of such argumentation. To preserve autonomy, one should not further weaken consent as legal ground for processing but improve transparency and individual control mechanisms, ia through software solutions.^179^

The processing might also be lawful without consent if it is necessary to protect the vital interest of the data subject or for the provision of a service explicitly requested by the user, limited to medical purposes, based on law or contract with a healthcare professional and subject to specific safeguards. However, the discussions above have shown that there are discrepancies between the necessity-grounds in the GDPR and the ePrivacy rules, generating legal insecurity. Only the cumulative application of the two instruments ensures adequate legal grounds for processing ICTIMD data. Thus, if the requirements of the PECD and the GDPR are aligned, it should, in our opinion, be a *two-way* alignment, keeping both the sector-specific limitations as well as the higher thresholds for health-related data. Finally, an explicit vital interest exception for content data should also be included in the EPR.

## Footnotes

1 See the definition of ‘medical devices’ art 2(1) in Regulation (EU) 2017/745 of the European Parliament and of the Council of 5 April 2017 on medical devices, amending Directive 2001/83/EC, Regulation (EC) No 178/2002 and Regulation (EC) No 1223/2009 and repealing Council Directives 90/385/EEC and 93/42/EEC [2017] OJ L117/1. Further, see Timo Minsen and others, ‘When Does Stand-Alone Software Qualify as a Medical Device in the European Union?’, (2020) 28(3) Medical Law Review 615–624.

2 For example, pacemakers, nerve stimulators and biosensors.

3 Matthew Barrett and others, ‘Artificial Intelligence Supported Patient Self-Care in Chronic Heart Failure: A Paradigm Shift from Reactive to Predictive, Preventive and Personalised Care’ (2019) 10 EPMA Journal 445, 448.

4 Farshad Firouzi and others, ‘From EDA to IoT eHealth: Promises, Challenges, and Solutions’ (2018) 37(12) IEEE Transactions on Computer-Aided Design of Integrated Circuits and Systems 2965, 2967.

5 Carmen Camara and others, ‘Security and Privacy Issues in Implantable Medical Devices: A Comprehensive Survey’ (2015) 55 Journal of Biomedical Informatics 272.

6 Firouzi (n 4) 2967.

7 Commission, ‘Proposal for a Regulation of the European Parliament and of the Council concerning the respect for private life and the protection of personal data in electronic communications and repealing Directive 2002/58/EC (Regulation on Privacy and Electronic Communications)’ COM/2017/010 final, Recital 20.

8 Directive 95/46/EC of 24 October 1995 on the protection of individuals with regard to the processing of personal data and on the free movement of such data [1995] OJ L281/31.

9 Regulation (EU) 2016/679 of 27 April 2016 on the protection of natural persons with regard to the processing of personal data and on the free movement of such data, and repealing Directive 95/46/EC [2016] OJ L119/1.

10 Consolidated Version of Directive 2002/58/EC of 12 July 2002 concerning the processing of personal data and the protection of privacy in the electronic communications sector [2009] OJ L201/37.

11 EPR (n 7).

12 See Muireann Quigley and Semande Ayihongbe, ‘Everyday Cyborgs: On Integrated Persons and Integrated Goods,’ (2018) 26(2) Medical Law Review 276.

13 Daniel Halperin and others, ‘Pacemakers and Implantable Cardiac Defibrillators: Software Radio Attacks and Zero-Power Defenses’ [2008] Proceedings—IEEE Symposium on Security and Privacy 129.

14 MDR (n 1) arts 62(4)(h), 72(3), 92(4), 110(1) and (2).

15 GDPR art 4(1).

16 ibid.

17 Eleni Kosta and Diana M Bowman, ‘Implanting Implications: Data Protection Challenges Arising from the Use of Human ICT Implants’ in Diana M Gasson and others (eds), *Human ICT Implants: Technical, Legal and Ethical Considerations* (TMC Asser Press 2012) 102.

18 GDPR Recital 26.

19 See ia Mostafa Langarizadeh and others, ‘Effectiveness of Anonymization Methods in Preserving Patients' Privacy: A Systematic Literature Review’ (2019) 248(6) Studies in Health Technology and Informatics, 80-87.

20 GDPR Recital 26.

21 ibid.

22 GDPR Recital 30.

23 Bradley D Nelson and others, ‘Wireless Technologies for Implantable Devices’ (2020) 20 Sensors, 4604. <https://doi.org/10.3390/s20164604>.

24 Kosta and Bowman (n 17) 102.

25 For example, Case C-101/01 *Lindqvist* [2003] ECR I-12971 para 50 (regarding the DPD, but valid for the GDPR).

26 See EPR art 4(3)c for definition of ‘metadata’.

27 ia EDPB, ‘Guidelines 03/2020 on the Processing of Data Concerning Health for the Purpose of Scientific Research in the Context of the COVID-19 Outbreak’ (2020), version 1.1, 5. <https://edpb.europa.eu/our-work-tools/our-documents/guidelines/guidelines-032020-processing-data-concerning-health-purpose_en>.

28 Griet Verhenneman, *The Patient, Data Protection and Changing Healthcare Models: The Impact of e-Health on Informed Consent, Anonymisation and Purpose Limitation* (Intersentia 2021) 343.

29 The Norwegian Consumer Council, ‘Health Data for Sale? Consumer Protection and Privacy in Blood Pressure Monitors and Blood Glucose Metres for Home Use’ (2017) 6. <https://www.forbrukerradet.no/wp-content/uploads/2017/09/2017-09-06-report-privacy-eng.pdf>.

30 GDPR Recital 15. Pursuant to art 2(1), it applies to processing wholly or partly by automated means.

31 Kosta and Bowman (n 17) 104.

32 See also C-342/12 *Worten Equipamentos para o Lar SA v Autoridade para as Condições de Trabalho (ACT)* [2013] ECLI:EU:C:2013:355 para 20 where the CJEU describe the collection, organization, storage, consultation, use, and transmission of data as ‘*the* processing of personal data’.

33 GDPR Recitals 50 and 32.

34 Serge Gutwirth, *Privacy and the Information Age* (Rowman & Littlefield Publishers 2002) 97, and for further discussion covering the GDPR, see Andreas G Meyer, ‘Identifying Controllers and Processors Pursuant to the General Data Protection Regulation’ (2018) 4 CompLex 16.

35 GDPR art 35(1).

36 See Marco Todde and others, ‘Methodology and Workflow to Perform the Data Protection Impact Assessment in Healthcare Information Systems’ (2020) 19 Informatics in Medicine Unlocked, 100361. <https://doi.org/10.1016/j.imu.2020>.

37 Case C-210/16 *Unabhängiges Landeszentrum für Datenschutz Schleswig-Holstein v Wirtschaftsakademie Schleswig-Holstein GmbH* [2018] ECLI:EU:C:2018:388.

38 PECD art 1(1).

39 Consolidated Version of Directive (EU) 2018/1972 of 11 December 2018 establishing the European Electronic Communications Code (EECC) (Recast) PE/52/2018/REV/1 [2018] OJ L321/36.

40 EPR art 2(1).

41 EPR art 4(3)(a).

42 EPR art 4(3)(b) and (c), Recital 2.

43 Consolidated version of the Treaty on the Functioning of the European Union [2012] OJ C326/47 (TFEU).

44 Case C-263/86 *Belgian State v René Humbel and Marie-Thérèse Edel* ECR [1988] 05365 para 17.

45 Case C-281/06 *Hans-Dieter Jundt and Hedwig Jundt v Finanzamt Offenburg* [2007] ECR I-12246 para 33.

46 Case C-352/85 *Bond van Adverteerders v Netherlands State* [1988] ECR 2085.

47 PECD Recital 24.

48 Directive 2008/63/EC of 20 June 2008 on competition in the markets in telecommunications terminal equipment, [2008] OJ L162/20.

49 Yves Poullet, ‘About the E-Privacy Directive: Towards a Third Generation of Data Protection Legislation?’ in Serge Gutwirth and others (eds), *Data Protection in a Profiled World* (Springer 2010) 18, fn 39.

50 Kosta and Bowman (n 17) 109.

51 Nelson and others (n 23) 3.

52 EECC art 2(8).

53 European Council, Proposal for a Regulation of the European Parliament and of the Council concerning the respect for private life and the protection of personal data in electronic communications and repealing Directive 2002/58/EC (Regulation on Privacy and Electronic Communications) Interinstitutional File: 2017/0003(COD) 5008/21 [2021]

54 ibid Annex I.

55 McGraw-Hill and Sybil Parker, *McGraw-Hill Dictionary of Scientific & Technical Terms* (6th edn, The McGraw-Hill Companies Inc 2002).

56 WP29, ‘Opinion 2/2008 on the Review of the Directive 2002/58/EC on Privacy and Electronic Communications (e-Privacy Directive)’ (WP150 2008) 4. <https://ec.europa.eu/justice/article-29/documentation/opinion-recommendation/files/2008/wp150_en.pdf>.

57 On ‘extranet’, see Thierry de Gorguette d’Argoeuves, ‘Extranet or the Too-Little-Known Linchpin of Globalization’ (2010) 3(1) AU-GSB e-JOURNAL 13. <http://www.assumptionjournal.au.edu/index.php/AU-GSB/article/view/404/356>

58 Refers to a network on top of another network. This can be implemented for cybersecurity purposes locally, but the term may also cover structures on the Internet as such.

59 Fog Computing refers to systems where only the edges of an infrastructure, such as routers and other entry points into a network, make a substantial amount of the calculations and general computations.

60 See Amir M Rahmani, ‘Exploiting Smart e-Health Gateways at the Edge of healthcare Internet-of-Things: A Fog Computing Approach’ (2018) 78(2) Future Generation Computer Systems 641.

61 EDPS Opinion on the Proposal for a Regulation on Privacy and Electronic Communications (ePrivacy Regulation), Opinion 6/2017 13.

62 WP29, ‘Opinion 1/2010 on the Concepts of "Controller" and "Processor"’ (WP169 2010) 19. <https://ec.europa.eu/justice/article-29/documentation/opinion-recommendation/files/2010/wp169_en.pdf>.

63 WP29, ‘Opinion 8/2014 on the Recent Developments on the Internet of Things’ (WP223 2014)11. <https://ec.europa.eu/justice/article-29/documentation/opinion-recommendation/files/2014/wp223_en.pdf>.

64 GDPR art 4(8).

65 Alex Tolsma, ‘GDPR and the Impact on Cloud Computing’ (Deloitte Undated). <https://www2.deloitte.com/nl/nl/pages/risk/articles/cyber-security-privacy-gdpr-update-the-impact-on-cloud-computing.html> accessed 26 July 2022.

66 More on this: EDPB, ‘Guidelines 07/2020 on the Concepts of Controller and Processor in the GDPR’ (2020). <https://edpb.europa.eu/our-work-tools/our-documents/guidelines/guidelines-072020-concepts-controller-and-processor-gdpr_en>.

67 For more on this, see Antonio Kung and others, ‘A Privacy Engineering Framework for the Internet of Things’ in Ronald Leenes and others (eds), *Data Protection and Privacy: (In)visibilities and Infrastructures* (Springer 2017) 166.

68 GDPR art 28(10).

69 WP223 (n 63) 14

70 WP29, ‘Opinion 02/2013 on Apps on Smart Devices’ (WP202 2013) 7. <https://ec.europa.eu/justice/article-29/documentation/opinion-recommendation/files/2013/wp202_en.pdf>.

71
*The EPR v 5008/21*.

72 ibid.

73 GDPR art 5(1).

74 Verhenneman (n 28) 160.

75 ibid.

76 ibid.

77 See also GDPR Recital 51.

78 Verhenneman (n 28) 160.

79 EDPS Opinion 6/2017 (n 61)a 3.

80 PECD Recital 14.

81 See the *EPR v 5008/21* Recital (20aaa) and Part III, amendments to the Text, para 40.

82 WP223 (n 63) 14 and the *EPR v 5008/21* Recital (20aa).

83 The principle ‘lex specialis derogat legi generali’ implies that special provisions prevail over general rules in situations which they specifically regulate. See Joined Cases T-60/06 *RENV II and T-62/06 RENV II Italian Republic v European Commission* [2016] ECLI:EU:T:2016:233 para 81.

84 EDPB, ‘Opinion 5/2019 on the Interplay between the ePrivacy Directive and the GDPR, in Particular Regarding the Competence, Tasks and Powers of Data Protection Authorities’ (2019) 13. See also GDPR art 95 and Recital 173. <https://edpb.europa.eu/our-work-tools/our-documents/opinion-board-art-64/opinion-52019-interplay-between-eprivacy_en>.

85 ibid. 13.

86 ibid. 14.

87 ibid.

88 EPR Recital 5.

89 ia, EDPB, ‘Guidelines 01/2020 on Processing Personal Data in the Context of Connected Vehicles and Mobility Related Applications’ V2.0 (2021) 7. <https://edpb.europa.eu/our-work-tools/our-documents/guidelines/guidelines-012020-processing-personal-data-context_en>.

90 ibid. 8.

91 As also, eg illustrated in EDPB, ‘Guidelines 04/2020 on the Use of Location Data and Contact Tracing Tools in the Context of the COVID-19 Outbreak’ (2020). <https://edpb.europa.eu/our-work-tools/our-documents/guidelines/guidelines-042020-use-location-data-and-contact-tracing_en>.

92 Verhenneman (n 28) 137 and 141.

93 PECD art 2(f) and Recital 17 and the EPR art 9(1).

94 GDPR art 7(3) and Recital 42.

95 GDPR Recital 43.

96 Verhenneman (n 28) 150.

97 WP29, ‘Working Document on the Processing of Personal Data Relating to Health in Electronic Health Records (EHR)’ (WP131 2007) 8. <https://ec.europa.eu/justice/article-29/press-material/public-consultation/ehr/2007_ehr/ms-national/dept_health_and_children_ie_en.pdf>.

98 ibid.

99 EDPB, ‘Opinion 3/2019 Concerning the Questions and Answers on the Interplay between the Clinical Trials Regulation (CTR) and the General Data Protection Regulation (GDPR)’ (2019) para 20. <https://edpb.europa.eu/our-work-tools/our-documents/opinion-art-70/opinion-32019-concerning-questions-and-answers_en>.

100 WP29, ‘Opinion 15/2011 on the Definition of Consent’ (WP187 2011) 9. <https://ec.europa.eu/justice/article-29/documentation/opinion-recommendation/files/2011/wp187_en.pdf>.

101 ibid.

102 Verhenneman (n 28) 181.

103 Case C-673/17, *Bundesverband der Verbraucherzentralen und Verbraucherverbände Verbraucher-zentrale Bundesverband eV v Planet49 GmbH* [2019] ECLI:EU:C:2019:801 para 74. 

104 GDPR art 13(2), art 5 and Recital 60.

105 GDPR art 13(2)(f). The nature of this requirement is extensively discussed in the academic literature. See ia Maja Brkan, ‘Do Algorithms Rule the World? Algorithmic Decision-Making and Data Protection in the Framework of the GDPR and Beyond’ (2019) 27 International Journal of Law and Information Technology 91.

106 Ronan Hamon and other, ‘Bridging the Gap Between AI and Explainability in the GDPR: Towards Trustworthiness-by-Design in Automated Decision-Making’ (2022) IEEE Computational Intelligence Magazine, 17(1), 74; Brkan (n 105) 113.

107 See ia Verhenneman (n 28) 164.

108 OECD, *The OECD Privacy Framework* (OECD 2013) 67 and WP223 (n 63) 6.

109 WP223 (n 63) 4.

110 The Norwegian Consumer Council (n 29) 8.

111 WP29, ‘Guidelines on Transparency under Regulation 2016/679’ (WP260 2017) 7. <https://ec.europa.eu/newsroom/article29/items/622227/en>.

112 Hamon (n 106).

113 ibid.

114 ibid.

115 Proposal for a Regulation of the European Parliament and of the Council concerning the respect for private life and the protection of personal data in electronic communications and repealing Directive 2002/58/EC (Regulation on Privacy and Electronic Communications) Interinstitutional File: 2017/0003(COD) 6087/21 [2021] art 6(2).

116 EDPB Guidelines 05/2020 on consent para 56.

117 GDPR Recital 32.

118 WP29, ‘Opinion 3/2013 on Purpose Limitation’ (WP203, 2013) 16.

119 WP131 (n 97) 8; WP187 (n 100) 18.

120 Discovery-driven approach refers to connecting and using existing IoT devices in a given area in analysis or use. For more on this, see Dimitrios Georgakopoulos and others, ‘Discovery-Driven Service Oriented IoT Architecture’ (2015) IEEE Conference on Collaboration and Internet Computing 142, doi:10.1109/CIC.2015.34.

121 The focus on broad purposes is to justify and use as many sources of data as possible, see ibid. for specific comments on this. See also Verhenneman (n 28) 182.

122 WP29, ‘Joint Contribution to the Consultation of the European Commission on the Legal Framework for the Fundamental Right to Protection of Personal Data, The future of Privacy’ (WP168 2009) 17. <https://ec.europa.eu/justice/article-29/documentation/opinion-recommendation/files/2009/wp168_en.pdf>.

123 Also called ‘tiered’, ‘layered’, and ‘participatory’ consent—Verhenneman (n 28) 197.

124 Proposal for a Regulation of The European Parliament and of The Council on the protection of individuals with regard to the processing of personal data and on the free movement of such data (General Data Protection Regulation) [First reading] LIMITE 15039/25, 15 December 2015 as interpreted by Verhenneman (n 28) 161.

125 GDPR art 7 and Recital 42.

126 WP29, ‘Guidelines on Consent under Regulation 2016/679’ (WP259 rev. 01 2017) para 93. <https://ec.europa.eu/newsroom/article29/items/623051/en>.

127 See ia ibid para 97.

128 ibid para 94.

129
*Draft EPR v 6087/21* art 4a(2aa) and Recital 20a.

130 EDPB, ‘Statement of the EDPB on the Revision of the ePrivacy Regulation and Its Impact on the Protection of Individuals with Regard to the Privacy and Confidentiality of Their Communications’ (2018) s 5. <https://edpb.europa.eu/our-work-tools/our-documents/other/statement-edpb-revision-eprivacy-regulation-and-its-impact_en>.

131 WP260 (n 111) para 9.

132 WP259 (n 126) para 107.

133 ibid para 111.

134 ibid para 110.

135 Paul Quinn and others, ‘The Data Protection and Medical Device Frameworks—Obstacles to the Deployment of mHealth across Europe?’ (2013) 20 European Journal of Health Law, 185-204 198.

136 The Norwegian Consumer Council (n 29) 8.

137 EDPB, ‘Guidelines 2/2019 on the Processing of Personal Data under Article 6(1)(b) GDPR in the Context of the Provision of Online Services to Data Subjects’ (2019) para 23. <https://edpb.europa.eu/our-work-tools/our-documents/guidelines/guidelines-22019-processing-personal-data-under-article-61b_en>.

138 ibid para 25.

139 Case C-524/06, *Heinz Huber v. Bundesrepublik Deutschland*, [2008] I-09705 para 52.

140 European Commission, *ePrivacy Directive: Assessment of Transposition, Effectiveness and Compatibility with proposed Data Protection Regulation* (European Commission 2015, SMART 2013/0071) 60.

141 ibid.

142 ibid.

143 EDPB (n 91) para 35.

144 Quinn (n 135) 198.

145 WP131 (n 97) 10 and 11.

146 Christopher Kuner and Ludmila Georgieva, ‘Article 9 Processing of Special Categories of Personal Data’ in Kuner and others (eds), *The EU General Data Protection Regulation (GDPR): A Commentary* (Oxford Scholarship Online 2021) 380.

147 Trix Mulder, ‘Health Apps, Their Privacy Policies and the GDPR’ (2019) 10(1) European Journal of Law and Technology 6.

148 GDPR Recital 46.

149 Draft EPR Jan 2021 art 8(f).

150 See ch VI. B.

151 European Commission (n 140) 60.

152 ia GDPR Recital 10.

153 Pawel Rotter and others, ‘Implantable Medical Devices: Privacy and Security Concerns’ in Mark N. Gasson, Eleni Kosta, Diana M. Bowman (eds), *Human ICT Implants: Technical, Legal and Ethical Considerations* (TMC Asser Press 2012) 65.

154 WP29, ‘Opinion 01/2017 on the Proposed Regulation for the ePrivacy Regulation (2002/58/EC)’ (WP247 2017) 15. See also EDPS Opinion 2017/6, ‘EDPS Opinion on the Proposal for a Regulation on Privacy and Electronic Communications (ePrivacy Regulation)’ 16 and 20. <https://edps.europa.eu/sites/edp/files/publication/17-04-24_eprivacy_en.pdf>.

155 Case F-46/09, *V v European Parliament* ECLI:EU:F:2011:101, para 123; TFEU art 16(1); Consolidated Version of the Treaty on European Union [2008] OJ C 115/13 art 6; Case C-311/18, *Data Protection Commissioner v Facebook Ireland Limited and Maximillian Schrems* [2020] ECLI:EU:C:2020:559 para 169.

156 Charter of Fundamental Rights of the European Union [2000] OJ 364/1.

157 Charter art 52(1) as interpreted in Case C-311/18 (n 155) para 174.

158 ia GDPR Recital 4.

159 Rolf H Weber and Romana Weber, *Internet of Things: Legal Perspectives* (Springer 2019) 41.

160 As first formulated in Bundesverfassungsgericht, 15.12.1983, Volkszählungsurteil, BVerfGE Bd. 65, s 1. Further elaborated by the European Court of Human Rights in its case law and Guide on art 8 of the European Convention on Human Rights, Updated on 31 December 2020 para 180.

161 Charter art 3(2).

162 EPR Explanatory Memorandum paras 2.3 and 3.1.

163 OECD, ‘Privacy Expert Group Report on the Review of the 1980 OECD Privacy Guidelines’ (2013) OECD Digital Economy Papers No 229, OECD Publishing, Paris, 8.

164 ia Ann Cavoukian, ‘Evolving FIPPs: Proactive Approaches to Privacy, Not Privacy Paternalism’ in Serge Gutwirth, Ronald Leenes and Paul de Hert (eds), *Reforming European Data Protection Law* (Law, Governance and Technology Series 20, 2015).

165 ibid.

166 See ia GDPR art 5, 13, and 14.

167 As ia enshrined in the Charter arts 2 and 35.

168 ia GDPR Recital 7.

169 TFEU art 288(2).

170 ia Orange, ‘Committed to Europe, The ePrivacy Draft Regulation, Protecting Privacy in Europe’ (2017) <https://www.orange.com/sites/orangecom/files/documents/2020-10/Orange%20views%20on%20the%20ePrivacy%20Regulation%20-%20May%202017.pdf> accessed 26 July 2022; Interactive Advertising Bureau (IAB) Europe, Position on the Review of the ePrivacy Directive, (2016) 6.

171 More on this in the EPR Explanatory memorandum 6.

172 CERRE, ‘Consumer Privacy in Network Industries, a CERRE Policy Report’ (2016) 6. <http://old.iabeurope.eu/wp-content/uploads/2017/06/20170328-IABEU-ePR_Position_Paper.pdf>.

173 ia Nicolas Terry, ‘Protecting Patient Privacy in the Age of Big Data’ (2013) 4 Indiana University Robert H McKinney School of Law Research Paper, doi:10.2139/ssrn 2153269.

174 WP247 (n 154) 6. European Digital Rights (EDRi), ‘e-Privacy Directive: Frequently Asked Questions’ (EDRi 2016) <https://edri.org/our-work/epd-faq/> accessed 26 July 2022.

175 WP247 (n 154) 3.

176 Directorate General for Internal Policies, ‘An assessment of the Commission’s Proposal on Privacy and Electronic Communications’ (2017) PE 583.152 20.

177 Proposal for a Regulation on the European Health Data Space (COM(2022) 197/2. We note that if implemented in its current form, it will allow expanded use of health data. It may also lead to a hard regulation of specifically software, which in its literal wording is a first for the EU in a technologically specific manner. As this is a proposal at an early stage, however, it will be given limited space in this article. However, as it builds on the possibilities in the GDPR for a specific EU legislation on the use of personal electronic health data, there is reason to believe that many of the perspectives covered here will be relevant also after its implementation.

178 See also European Commission, ‘Assessment of the EU Member States’ Rules on Health Data in the Light of the GDPR’ (2021).

179 Cavoukian (n 164).

